# Whey Proteins Reduce Appetite, Stimulate Anorexigenic Gastrointestinal Peptides and Improve Glucometabolic Homeostasis in Young Obese Women

**DOI:** 10.3390/nu11020247

**Published:** 2019-01-23

**Authors:** Antonello E. Rigamonti, Roberto Leoncini, Claudia Casnici, Ornella Marelli, Alessandra De Col, Sofia Tamini, Elisa Lucchetti, Sabrina Cicolini, Laura Abbruzzese, Silvano G. Cella, Alessandro Sartorio

**Affiliations:** 1 Department of Clinical Sciences and Community Health, University of Milan, via Vanvitelli 32, 20129 Milan, Italy; silvano.cella@unimi.it; 2 Department of Medical Biotechnologies, University of Siena, 53100 Siena, Italy; roberto.leoncini@unisi.it; 3 Ferdinando Santarelli Foundation, 20122 Milan, Italy; claudia.casnici@gmail.com; 4 Department of Medical Biotechnologies and Translational Medicine, University of Milan, 20133 Milan, Italy; ornella.marelli@unimi.it; 5 Istituto Auxologico Italiano, IRCCS, Experimental Laboratory for Auxo-endocrinological Research, 28824 Piancavallo (VB), Italy; a.decol@auxologico.it (A.D.C.); sofia.tamini@gmail.com (S.T.); e.lucchetti@auxologico.it (E.L.); sabrinacicolini92@gmail.com (S.C.); l.abbruzzese@auxologico.it (L.A.); sartorio@auxologico.it (A.S.); 6 Istituto Auxologico Italiano, IRCCS, Division of Auxology and Metabolic Diseases, 28824 Piancavallo (VB), Italy

**Keywords:** whey proteins, maltodextrins, obesity, appetite, anorexigenic gastrointestinal peptides, insulin, glucose, amino acids

## Abstract

Introduction: Proteins, particularly whey proteins, represent the most satiating macronutrient in animals and humans. A dietetic regimen based on proteins enriched preload before eating might be a strategy to counteract obesity. Aims and Methods: The aim of the present study was to evaluate the effects of an isocaloric drink containing whey proteins or maltodextrins (preload) on appetite (satiety/hunger measured by a visual analogue scale or VAS), glucometabolic control (blood glucose/insulin), and anorexigenic gastrointestinal peptides (pancreatic polypeptide or PP, glucagon-like peptide 1 or GLP-1 and peptide YY or PYY) in a cohort of obese young women (*n* = 9; age: 18.1 ± 3.0 years; body mass index, BMI: 38.8 ± 4.5 kg/m^2^). After two and a half hours, they were administered with a mixed meal at a fixed dose; satiety and hunger were measured by VAS. Results: Each drink significantly augmented satiety and reduced hunger, and the effects were more evident with whey proteins than maltodextrins. Similarly, there were significant increases in GLP-1 and PYY levels (but not PP) after the ingestion of each drink; these anorexigenic responses were higher with whey proteins than maltodextrins. While insulinemia identically increased after each drink, whey proteins induced a lower glycemic response than maltodextrins. No differences in satiety and hunger were found after the meal, which is presumably due to the late administration of the meal test, when the hypophagic effect of whey proteins was disappearing. Conclusions: While whey proteins actually reduce appetite, stimulate anorexigenic gastrointestinal peptides, and improve glucometabolic homeostasis in young obese women, further additional studies are mandatory to demonstrate their hypophagic effects in obese subjects, when administered as preload before eating.

## 1. Introduction

The increasing prevalence of obesity in Western and also incoming countries has prompted the search for “bioactive” foods capable of satiating and reducing energy intake, thus determining an improvement of glucometabolic homeostasis [1,2].

Several studies have shown that proteins represent the most satiating macronutrient, and in particular, whey proteins are more satiating than carbohydrates (e.g., maltodextrins) and proteins of different sources [3]. In this context, in normal-weight subjects, a preload containing whey proteins (in comparison to maltodextrins), orally administered up to 2 h before, has been reported to reduce the subsequent ad libitum food intake, an effect associated with an increase in satiety and in some anorexigenic gastrointestinal peptides, including glucose-dependent insulinotropic polypeptide (GIP), cholecystokinin (CCK), pancreatic polypeptide (PP), and glucagon-like peptide 1 (GLP-1) [4,5,6]. Some authors suggest that digestion of whey proteins into the gastrointestinal tract provokes the rapid absorption of bioactive peptides and branched-chain amino acids (BCAAs), particularly leucine, in the local gastrointestinal wall or in the systemic blood stream, which, respectively, stimulates secretion of anorexigenic gastrointestinal peptides from specific enteroendocrine cells and act at the hypothalamic level on neurons regulating energy intake or expenditure [7]. As demonstrated in animal studies, these molecular and cellular effects increase satiety, stimulate thermogenesis, and reduce food intake [8].

Maltodextrins, although representing an energy source promptly usable in the post-exercise recovery phase [9], are characterized, when compared to whey proteins, by a high glycemic index, which, biochemically, can worsen glucose intolerance in an overweight or obese subject [10]. It is notable that there is evidence that daily consumption of milk-derived products, including whey proteins, can improve glycemic control in type 2 diabetic patients [11]. 

Some epidemiological studies have shown that the spread use of sugar-sweetened beverages has contributed to an increase in pediatric obesity worldwide [12,13]; furthermore, we have long known that an obese adolescent will easily become an obese adult [14]. Thus, it is important to invest in health prevention dedicated to younger people, particularly, children/adolescents and young adults, adopting, in an early phase, educational programs encompassing more appropriate lifestyles and dietetic regimes. The replacement of “obesiogenic” foods with others, hypocaloric, but satiating, may represent a valid strategy to counteract pediatric obesity [15].

Unfortunately, only few clinical researches have evaluated the effects of whey proteins in young obese subjects [16,17,18], who, in comparison to the normal-weight counterpart, present complex alterations in central and peripheral regulatory systems of food intake, including anorexigenic gastrointestinal peptides [19,20,21,22,23,24,25].

Based on the previous considerations, the aim of the present study was to evaluate, in a group of young obese women, the effects of a drink containing whey proteins or maltodextrins on satiety and hunger, secretion of some anorexigenic gastrointestinal peptides in circulation, particularly GLP-1, PP, and PYY, and glucometabolic homeostasis. Our hypothesis is that a preload of whey proteins, compared with maltodextrins, might be capable of reducing appetite (satiety and hunger), with an ensuing mixed lunch consumed two and a half hours later.

## 2. Materials and Methods

### 2.1. Patients and Experimental Protocol

Nine obese young women (age: 18.1 ± 3.0 years; body mass index, BMI: 38.8 ± 4.5 kg/m^2^; fat-free mass, FFM: 55.0 ± 5.8%; and fat mass, FM: 45.0 ± 5.8%) were recruited among patients hospitalized for a body weight reduction program at Istituto Auxologico Italiano, Piancavallo (VB). The study was completed before starting this program in order to avoid any carry-over effect due to weight, diet, and physical activity changes. Subjects having any disease, apart from morbid obesity, or subjects who take any drugs were excluded. Furthermore, weight should be stable (not more than 5 kg in the previous month) to take part in the study. All women were eumenorrheic.

**Figure 1:**
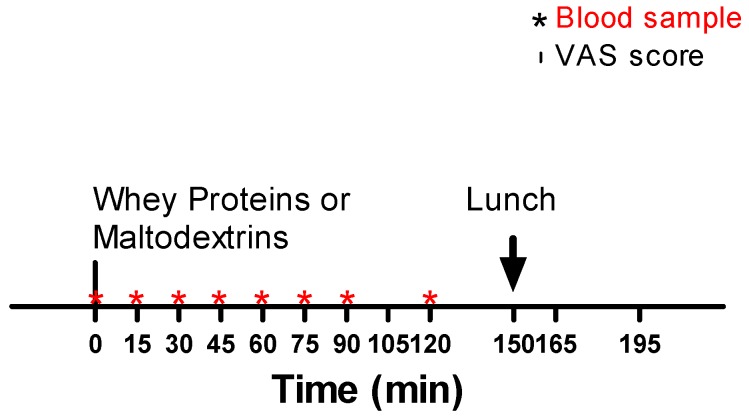
Overview of the experimental protocol.

The protocol has been summarized in Figure 1.

In separate days, with a wash-out period of at least 7 days, in agreement with a randomized order and cross-over design, starting from 8:00 a.m., the participants underwent two tests consisting of the oral administration, after 12 h of overnight fasting, of a drink containing whey proteins (45 g of Enervit Gymline Muscle 100% whey protein isolate cacao, Enervit spa, Erba, Italy, corresponding to 715 kj) or maltodextrin (43 g of Enervit Maltodestrine Sport, Enervit spa, Erba, Italy, corresponding to 715 kj), dissolved in 300 mL of semi-skimmed milk, corresponding to 585 kj, for a total of 1300 kj of metabolizable energy (ME). The drink was consumed within 15 min (100 mL every 5 min for three times). Each drink was prepared with the same color and taste (by using cacao powder) in order to avoid possible visual and taste conditioning. Blood samples were drawn from all participants starting from T0 (baseline, before administering the drink) until to T120, for a total of 7 samples, i.e., T0 (0 min), T15 (15 min), T30 (30 min), T45 (45 min), T60 (60 min), T90 (90 min), and T120 (120 min). By using a visual analogue scale (VAS), appetite (satiety and hunger) was evaluated at the following times: T0 (0 min), T15 (15 min), T30 (30 min), T45 (45 min), T60 (60 min), T75 (75 min), T90 (90 min), T105 (105 min), and T120 (120 min). In particular, subjects were asked to rate their satiety and hunger on a 10-cm line with labels at the extremities indicating the most negative and the most positive ratings.

After another half hour (i.e., at 150 min, T150), a mixed lunch (60% of carbohydrates, 30% of proteins, and 10% of fats, for a total of 2600 kj of energy intake, which was identical for both tests) was offered to all subjects and were asked to completely consume the meal within 15 min. Appetite (satiety and hunger) was evaluated, as previously described, at T150, T165 (165 min), and T195 (195 min). During the lunch, drinking still water was permitted (max 250 mL). 

### 2.2. Evaluation of Body Composition

Anthropometric characteristics were evaluated during the screening period. The evaluation of fat free mass (FFM) and fat mass (FM) was performed throughout bio-impedentiometry (Human-IM Scan, DS-Medigroup, Milan, Italy).

### 2.3. Blood Sampling and Biochemical Measurements

Blood was collected in tubes with or without anticoagulant (EDTA). Plasma or serum was separated by centrifugation and stored at −20 °C. 

The plasma PP level was determined by an ELISA kit P (Millipore, Saint Charles, MO, USA). The sensitivity was 12.3 pg/mL and the intra and inter-assay coefficients of variation (CVs) were 3.3% and 9.8%, respectively.

The total plasma PYY level, including both PYY_1-36_ and PYY_3-36_, was measured by an ELISA kit (Millipore, Saint Charles, MO, USA). The sensitivity was 6.5 pg/mL and the intra- and inter-assay CVs were 2.66% and 6.93%, respectively.

The total plasma GLP-1 level, including GLP-1_7-36 amide_, GLP-1_7-37_, GLP-1_9-36 amide_, GLP-1_9-37_, GLP-1_1-36 amide_, and GLP-1_1-37_, was determined by an ELISA kit (Millipore, Saint Charles, MO, USA). A DPP-IV (dipeptidyl protease IV) inhibitor (protease inhibitor cocktail, Sigma Aldrich-Merck, Darmstadt, Germany) was added to tubes (50 µl) in order to prevent GLP-1 breakdown. The sensitivity was 1.5 pmol/l and the intra and inter-assay CVs were 1% and <12%, respectively.

Serum insulin concentration was measured by a chemiluminescent immunometric assay (Immulite 2000, DPC, Los Angeles, CA, USA). The sensitivity was 2 µIU/mL and the intra and inter-assay CVs were 22–38% and 14–23%, respectively.

The serum glucose level was determined by the glucose oxidase enzymatic method (Roche Diagnostics, Monza, Italy).

### 2.4. Ethics Approval and Consent to Participate

The study protocol was approved by the Ethical Committee of Istituto Auxologico Italiano (research project code: 01C723; acronym: PROLATPEPOB), and all subjects (or their parents) gave their written consent after being fully informed about every aspect of the study protocol.

### 2.5. Statistical Analyses

The Sigma Stat 3.5 statistical software package (Systat Software, San Jose, CA, USA) was used for data analyses and GraphPad Prisma 5.0 software (GraphPad Software, San Diego, CA, USA) for data plotting. 

A power analysis was performed, a priori, to determine the sample size, considering that a difference in mean values of GLP-1 levels at T90 after whey proteins vs. maltodextrins was equal to 40.0 ± 15.0 pmol/l, with an α error of 0.05 at two tails and a power of 0.80.

The Shapiro-Wilk test showed that all parameters were normally distributed.

Results are reported as mean ± standard deviations (SD). 

A two-way ANOVA with repeated measures (with the two factors, time and group, and the interaction time × group), followed by the post hoc Tukey’s test, was used to compare all parameters within each experimental group (whey proteins or maltodextrins) over sampling times (intra-group analysis) and between the two experimental groups (whey proteins vs. maltodextrins) for any sampling time (inter-group analysis). The same statistical method was used to compare post-lunch responses of VAS scores for hunger and satiety vs. T150 (intra-group analysis) and among subjects administered with whey proteins vs. maltodextrins (inter-group analysis). 

Significance was set at a level of *p* < 0.05 for all the data.

## 3. Results

**Figure 2:**
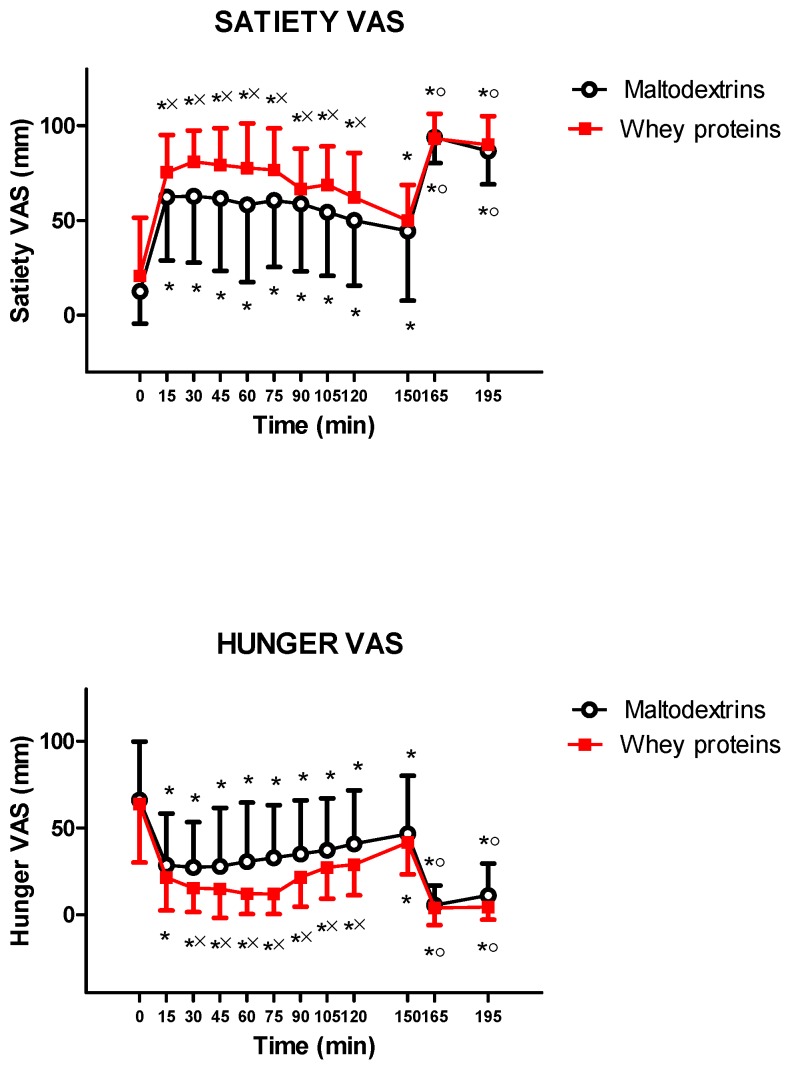
Changes of VAS (visual analogic scale) ratings of satiety (**top**) and hunger (**bottom**) in young obese subjects after the intake of a drink (completely within 15 min starting at T0), containing whey proteins or maltodextrins. At T150, a mixed lunch was offered and was completely consumed within 15 min. See the text for further details. Values are expressed as mean ± SD. The number of subjects was 9. * *p* < 0.05 vs. the corresponding T0 value; × *p* < 0.05 vs. the corresponding value of the maltodextrins-treated group; and ^○^ *p* < 0.05 vs. the corresponding T150 value. A two-way ANOVA with repeated measures (with the two factors time and group and the interaction time × group), followed by the post hoc Tukey’s test, was used.

The intake of isocaloric drinks containing whey proteins or maltodextrins significantly augmented and reduced satiety and hunger, respectively, (satiety: 0 min vs. 15, 30, 45, 60, 75, 90, 105, and 120 min for both drinks, *p* < 0.05; and hunger: 0 min vs. 15, 30, 45, 60, 75, 90, 105, and 120 min for both drinks, *p* < 0.05). Whey proteins induced more satiety and less hunger (satiety: *p* < 0.05 at 15, 30, 45, 60, 75, 90, 105, and 120 min vs. maltodextrins; and hunger: *p* < 0.05 at 30, 45, 60, 75, 90, 105, and 120 min vs. maltodextrins) (Figure 2).

There were the same significant effects of increased satiety and reduced hunger up to two and an half hours (T150) from the intake of each drink (vs. 0 min, *p* < 0.05), without any significant difference between the two experimental groups (whey proteins vs. maltodextrins). The following ingestion of a mixed lunch significantly increased satiety and reduced hunger (at 165 min and 195 min vs. 150 min, *p* < 0.05), without significant differences between the two experimental groups (whey proteins vs. maltodextrins) (Figure 2).

**Figure 3:**
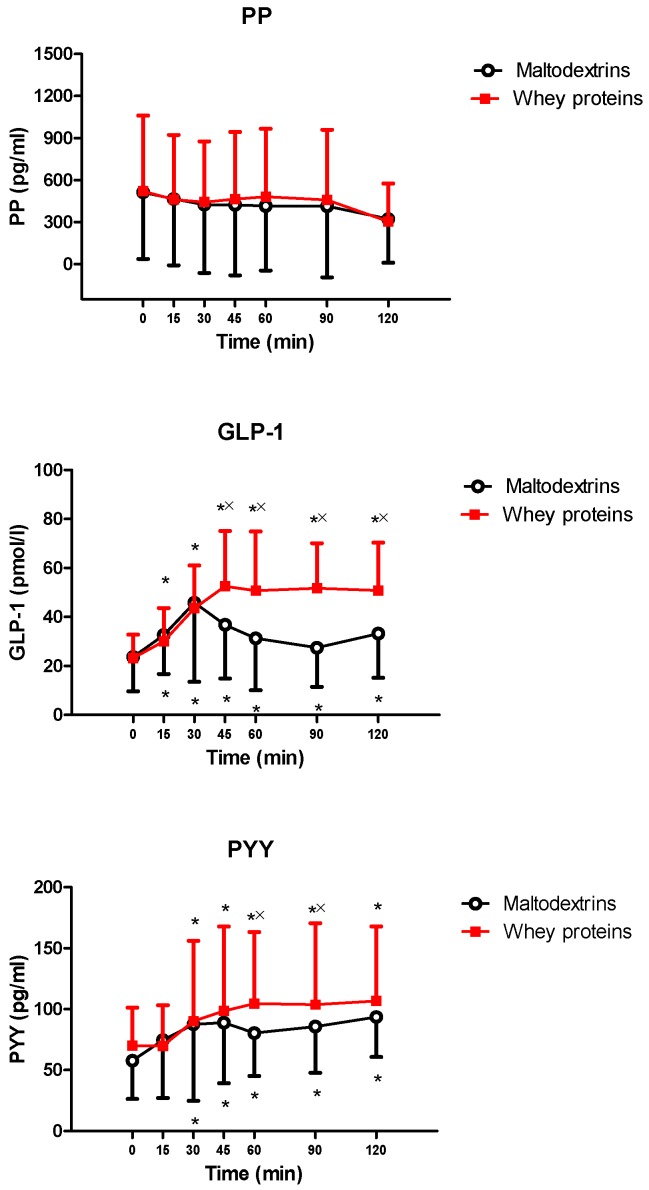
Changes of pancreatic polypeptide (PP) (**top**), glucagon-like peptide 1 (GLP-1) (**middle**), and peptide YY (PYY) (**bottom**) levels in young obese subjects after the intake of a drink (completely within 15 min starting at T0), containing whey proteins or maltodextrins. See the text for further details. Values are expressed as mean ± SD. The number of subjects was 9. * *p* < 0.05 vs. the corresponding T0 value; and × *p* < 0.05 vs. the corresponding value of the maltodextrins-treated group. A two-way ANOVA with repeated measures (with the two factors, time and group, and the interaction time × group), followed by the post hoc Tukey’s test, was used.

PP levels did not significantly change after the intake of each drink (vs. 0 min and between whey proteins vs. maltodextrins). On the contrary, the intake of each drink significantly increased GLP-1 levels (0 min vs. 15, 30, 45, 60, 90, and 120 min for both drinks, *p* < 0.05). Whey proteins induced higher GLP-1 levels (*p* < 0.05 at 45, 60, 90, and 120 min vs. maltodextrins). Furthermore, the intake of each drink significantly increased PYY levels (0 min vs. 30, 45, 60, 90, and 120 min for both drinks, *p* < 0.05). Whey proteins induced higher PYY levels (*p* < 0.05 at 60 and 90 min vs. maltodextrins) (Figure 3).

**Figure 4:**
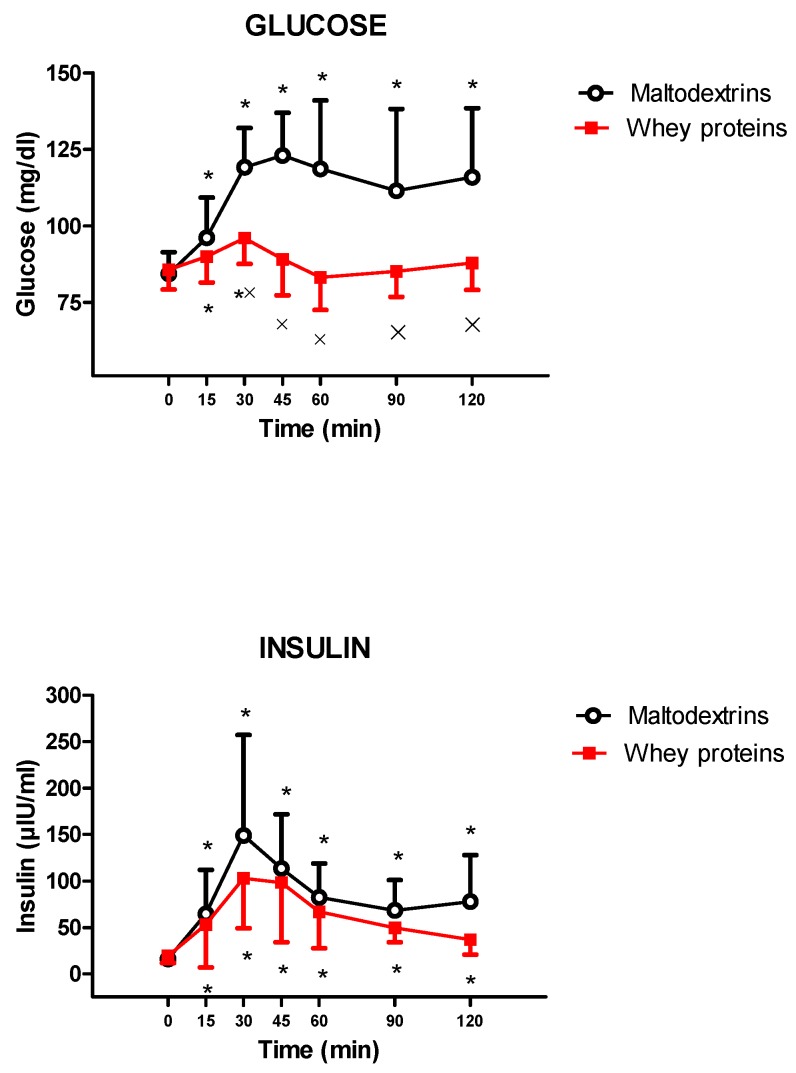
Changes of glucose (**top**) and insulin (**bottom**) levels in young obese subjects after the intake of a drink (completely within 15 min and starting at T0), containing whey proteins or maltodextrins. See the text for further details. Values are expressed as mean ± SD. The number of subjects was 9. * *p* < 0.05 vs. the corresponding T0 value; and × *p* < 0.05 vs. the corresponding value of the maltodextrins-treated group. A two-way ANOVA with repeated measures (with the two factors, time and group, and the interaction time × group), followed by the post hoc Tukey’s test, was used.

The intake of isocaloric drinks containing whey proteins or maltodextrins significantly increased glycaemia and insulinemia (glucose: 0 min vs. 15 and 30 min for that containing whey proteins and 15, 30, 45, 60, 90, and 120 min for that containing maltodextrins, *p* < 0.05; and insulin: 0 min vs. 15, 30, 45, 60, 90, and 120 min for both drinks, *p* < 0.05). Whey proteins induced a lower increase of glycaemia (glucose: *p* < 0.05 at 30, 45, 60, 90, and 120 min vs. maltodextrins), without any significant difference in insulinemic responses between the two experimental groups (whey proteins vs. maltodextrins) (Figure 4).

## 4. Discussion

Whey proteins only represent 20% of the total milk proteins; casein is the most abundant one. Whey proteins consist of a highly soluble mix of proteins, including β-lactoglobulin, α-lactalbumin, proteose peptone, immunoglobulins, bovine serum albumin, lactoperoxidase, and lactoferrin [26]. Another component of whey proteins is glycomacropeptide (GMP), which derives from the action of chymosin on casein [27]. The principal source of BCAAs in whey proteins is GMP [26]. Robust evidence supports that hypophagic, anorexigenic, and antidiabetogenic properties, attributed to whey proteins, are mediated by “bioactive peptides” (or single “bioactive amino acids”), which, generated from digestion, exert specific biochemical or pharmacological actions [8].

In the present study, whey proteins significantly induced more satiety and less hunger when compared to maltodextrins, which were effects that were already evident 30 min after the ingestion of the drink and persisted up to 120 min. The differences in satiety and hunger between the two experimental groups (i.e., whey proteins and maltodextrins) were no more significant at 150 min when the mixed lunch was offered to participants, thus explaining the similar post-prandial responses in satiety and hunger. The ineffectiveness of whey proteins to reduce appetite (more than maltodextrins) was likely due to the late consumption of the lunch. A more appropriate choice of time (i.e., before 120 min from intake of the drink) would have perhaps allowed us to observe a significantly lower appetite (i.e., increased satiety and decreased hunger) after whey proteins than maltodextrins [4,5]. Further studies are mandatory to confirm our hypothesis, possibly by adopting an experimental paradigm of ad libitum food intake.

The results of the present study confirm those already obtained in normal-weight adults [4,5,28,29], but are more valuable because, in comparison to other previous studies, whey proteins were administered to a young obese population, which were the most exposed to sociocultural or commercial “anti-health” messages, including the proposal of obesiogenic foods [30]. Therefore, despite the above-reported limitation of our study, a diet enriched with whey proteins (or other milk-derived bioactive peptides) could represent a valid strategy, contributing to counteract pediatric obesity.

In the present study, the intake of drinks containing whey proteins or maltodextrins significantly increased some anorexigenic peptides, such as PYY and GLP-1, but not others, such as PP. Importantly, secretions of PYY and GLP-1 were significantly higher after whey proteins than maltodextrins, a difference persisting up to 120 min (at least for GLP-1), the last time point of blood sampling, as stated by the study protocol. The changes in anorexigenic gastrointestinal peptides are congruent with those in appetite (satiety and hunger), as previously described. While other studies have reported that whey proteins stimulate anorexigenic gastrointestinal peptides [6,31], to the best of our knowledge, it is the first time that whey proteins were shown to increase PYY and GLP-1 levels in a young obese population, a response that was higher when compared to that of an isocaloric drink containing maltodextrins. This finding might suggest the opportunity of replacing “obesiogenic” foods with more satiating, but hypocaloric, foods for children and adolescents [32].

According to some cell and animal studies, bioactive peptides or amino acids, deriving from the digestion of whey proteins (and not proteins of different sources), would bind to specific receptors, particularly the so-called amino acid taste receptors [33], and stimulate specialized enteroendocrine cells, present into the gastrointestinal wall, which post-prandially respond by secreting anorexigenic gastrointestinal peptides (as K cells or L cells), including PYY and GLP-1 [8]. In this context, by using a murine tumor cell line (STC-1) as an in vitro model of enteroendocrine cells, some authors have demonstrated the ability of specific milk proteins, including peptides and amino acids, to release a wide range of appetite-regulating gastrointestinal peptides [34,35,36].

An alternative (or additional) mechanism might be represented by the absorption of bioactive amino acids, particularly BCAAs, in circulation, which have been shown to stimulate the secretion of some anorexigenic gastrointestinal peptides [7].

In comparison to the negative results of the present study, PP levels were higher after whey proteins than maltodextrins when administered to normal-weight women [6]. To date, we are unable to explain the reason of this discrepancy. In fact, conflicting post-prandial responses in PP levels in obese subjects have been reported in very few studies enrolling a control (i.e., normal weight) group [37,38,39]; furthermore, differences in the protocol or analytical method (with cross-reactivity for similar gastrointestinal peptides) should not be ruled out [40].

In the present study, there were similar insulinemic responses after whey proteins or maltodextrins; in any case, whey proteins induced a significantly lower glycemic response than maltodextrins. This finding suggests that a greater glucose-dependent secretion of insulin occurs after whey proteins than maltodextrins. This insulinotropic effect may be mediated by GLP-1 (or other incretins), potently stimulated by the intake of whey proteins, and/or specific amino acids, deriving from the digestion of whey proteins, such as BCAAs that, by activating specific intracellular signaling pathways (e.g., mTOR or AMPK), stimulate the secretion of insulin from pancreatic β-cells [7,41].

Taking into account the increasing spread of type 2 diabetes in the pediatric obese population in the last two decades [42], the potential antidiabetogenic properties of whey proteins, resembling those of the classic glucose lowering drugs, should be further investigated in the future [11,43]. In this context, the demonstration that bioactive peptides, endowed with a DPP-IV inhibitory property, naturally generated during the digestion of whey proteins, is extremely intriguing [44].

Before closing, some limitations of our study should be mentioned. First of all, we recruited only a limited number of obese subjects and all were of female gender. Second, we measured circulating levels of anorexigenic gastrointestinal peptides up to 120 min; therefore, we do not know the post-prandial responses of these peptides from T150 to T210 between the two experimental groups (whey proteins vs. maltodextrins). Third, other gastrointestinal peptides, not only anorexigenic, but also orexigenic (such as ghrelin), might be involved in the satiating and hypophagic effects of whey proteins. Finally, the measurement of circulating levels of amino acids (single, total and BCAAs) would have allowed us to identify amino acids implicated in the satiating effect of whey proteins or to rule out the direct role of amino acids, suggesting the existence of other biochemical or physiological mechanisms underlying the satiating effect of whey proteins.

## 5. Conclusions

In conclusion, whey proteins reduce appetite, stimulate anorexigenic gastrointestinal peptides, and improve glucometabolic homeostasis in young obese women. Nevertheless, further additional studies are mandatory to demonstrate their hypophagic effect and to hypothesize their potential alternative use (e.g., vs. sugar-sweetened beverages) to counteract the development of obesity in young populations.

## Abbreviations

BCAA	branched-chain amino acid
BMI	body mass index
CCK	cholecystokinin
CV	coefficient of variation
DPP-IV	dipeptidyl protease IV
FFM	fat-free mass
FM	fat mass
GIP	glucose-dependent insulinotropic polypeptide
GLP-1	glucagon-like peptide 1
GMP	glycomacropeptide
PP	pancreatic polypeptide
PYY	peptide YY
sd	standard deviation
VAS	visual analogic scale
